# Efficacy of TachoSil^® ^patches in controlling Dacron suture-hole bleeding after abdominal aortic aneurysm open repair

**DOI:** 10.1186/1749-8090-4-60

**Published:** 2009-11-04

**Authors:** Guido Bajardi, Felice Pecoraro, Domenico Mirabella

**Affiliations:** 1 Vascular Surgery Unit - University of Palermo, Via Liborio Giuffrè, 5 - 90100 Palermo, Italy

## Abstract

**Purpose:**

The aim of this study is evaluate the efficacy of TachoSil^® ^patches in controlling suture-hole bleeding after elective infrarenal abdominal aortic aneurysm (AAA) replacement with Dacron graft.

**Materials and methods:**

Patients undergoing elective replacement of infrarenal AAA with Dacron grafts were prospectively randomized to TachoSil^® ^patches (Group I) or standard compression with surgical swabs (Group II).

We evaluated time to haemostasis, blood loss during the operation, blood loss after cross-clamp removal, duration of operation, drain volume, requirement for blood transfusion and surgeons rating of efficacy.

**Results:**

Twenty patients were randomized (10 patients in each treatment Group). The mean time to haemostasis was 264 ± 127.1 s (range: 180-600 s) in Group I and 408 ± 159.5 s (range: 120-720 s) in Group II (p = 0.026); mean blood loss during the operation was 503.5 ± 20.7 cc (range: 474-545 cc) in Group I and 615.7 ± 60.3 cc (range: 530-720 cc) in Group II (p < 0.001); mean blood loss after cross-clamp removal was 26.5 ± 4 g (range: 22-34 g) in Group I and 45.4 ± 4.6 (range: 38-52 g) in Group II (p < 0.001) and mean drain volume was 116.7 ± 41.4 cc (range: 79-230 cc) in Group I and 134.5 ± 42.8 cc (range: 101-250 cc) in Group II (p = 0.034). There were no serious adverse events associated with use of TachoSil^® ^patches.

**Conclusion:**

For patients undergoing aortic reconstruction with Dacron grafts, TachoSil^® ^patches were found to be safe and effective for the control of suture-hole bleeding.

## Introduction

Suture-hole bleeding, during aortic surgery, represents a risk for the patient in terms of blood loss and prolongation of operation due to additional suturing with danger of iatrogenic stenosis. Although this complication is more relevant in the use of expanded polytetrafluoroethylene, it may occur also after Dacron graft replacement [1,2]. Typically suture-hole bleeding is managed by compression with surgical swabs and reversal of heparin. Other methods such as application of oxidised cellulose, gelatine sponge, different forms of collagen and glues have also been tried with variable success [3].

TachoSil^® ^(haemostatic surgical patch; Nycomed, Linz, Austria) is a fixed combination of a collagen matrix coated with the coagulation factors, human fibrinogen and human thrombin. TachoSil^® ^is indicated in adults for supportive treatment in surgery for improvement of haemostasis, to promote tissue sealing, and for suture support in vascular surgery where standard techniques are insufficient [4]. It is a ready-to-use and absorbable haemostatic dressing. The aim of the study was to compare the efficacy and safety of TachoSil^® ^patches against standard surgical compression to control the suture-hole bleeding from Dacron grafts in aortic surgery.

## Materials and methods

**Figure 1:**
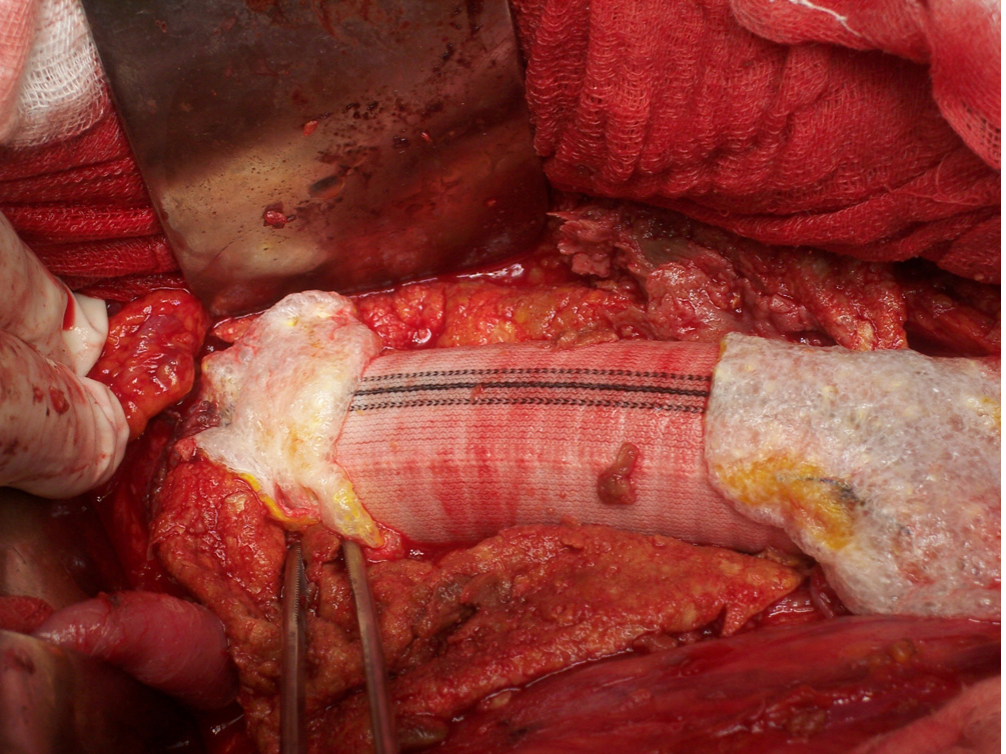
**Aorto-aortic Dacron straight tube and TachoSil^® ^application**.

Between June 2007 and June 2008 a total of 20 patients with intact infrarenal abdominal aortic aneurysm (AAA) were randomized in the same center. Mean age was 72.8 ± 6.1 years (range: 63-80 years) in the TachoSil^® ^Group (Group I) and 72.6 ± 4.5 years (range: 67-82 years) in the standard compression with surgical swabs Group (Group II). Mean transverse diameter was 7.1 ± 1.2 cm (range: 5.6-9 cm) in Group I and 6.8 ± 1 cm (range 5.5-8.6 cm) in Group II. Seventeen were male and 3 female (Table 1), 18 patients received an aorto-aortic Dacron straight tube (Fig 1) and two an aorto-bifurcated Dacron graft. All prosthesis used were Gelsoft™ Plus gelatin impregnated knitted graft (Vascutek, Terumo Company, Glasgow, Scotland). No patients were excluded after the randomization. Aortic and femoral anastomosis were evaluated. The anastomosis were performed using 2-0 or 5-0 prolene sutures. Systemic heparin (50-100 UI/Kg) was infused in aorto-bifurcated Dacron graft; in these two cases it hasn't be reversed. The local ethics committee approved the study, and informed written consent was obtained before inclusion from all patients. Allergy to any component of TachoSil^® ^was considered for exclusion prior the randomization. Patients, who did not require additional haemostatic measures on releasing the clamps, were also excluded from the study. Likewise patients with alteration of clotting parameters or liver diseases were excluded from the study. Sealed code envelope method was used to randomize patients to either treatment with TachoSil^® ^use or to standard compression with surgical swabs. The randomization code envelopes were opened just before the application of haemostatic measures. The nature of the treatments precluded blinding of the study.

**Table 1:** Patient demographics and comorbidity conditions.

**Factor**	TachiSil^®^, n (%)*	Control, n (%)*	**P**
Age, mean years	72.8 (63-80)	72.6 (67-82)	NS
Male gender	8 (80)	9 (90)	NS
Smoking history	4 (4	5	NS
Hypertension	6 (60)	5 (50)	NS
Diabetes Mellitus	0 (0)	1 (10)	NS
COPD	2 (20)	3 (30)	NS
CAD	0 (0)	0 (0)	NS
Cerebrovascular disease	0 (0)	0 (0)	NS
Renal Failure	0 (0)	0 (0)	NS
Aneurysm size, median cm	7.1 (5.6-9)	6.8 (5.5-8.6)	NS

COPD: chronic obstructive pulmonary disease; CAD: coronary artery disease

* Categoric data presented as number (%), continous data presented with the range

TachoSil^® ^patches were applied in Group I after moistening with physiological saline after clamp removing. Dosage (the number of patches used) depended on the size of the area to be covered. One patch of TachoSil^® ^is 9.5 × 4.8 × 0.5 cm in size. It contains a fixed combination of a collagen matrix coated with the coagulation factors, human fibrinogen (5.5 mg/cm^2^) and human thrombin (2.0 IU/cm^2^). TachoSil is produced by Nycomed (Linz, Austria), and is a further development of the TachoComb^® ^and TachoComb H^® ^products and is free of bovine components.

The moistened patch of TachoSil^® ^was applied with the yellow active side onto the wound surface after blood and other fluids were cleaned from the wound area. The patch had to extend around the anastomosis and left in loco for 3 min. After this period haemostasis was assessed. If haemostasis was not achieved by the time of this first look another 2 min of compression was applied. If haemostasis was still insufficient, the first patch was replaced with a new and left in loco for a maximum of 5 min before considering other methods. If a patch became displaced by bleeding during the first 5 min, it was replaced with a new piece. Patients in the control arm received compression with 10 × 10 cm surgical swabs. If haemostasis was suboptimal at the end of 10 min then other methods were adopted as necessary.

The main outcome measured was time to achieve haemostasis at the suture line. Haemostasis was defined as the time when the abdominal or inguinal wound was completely dry or sufficiently dry to complete the operation without additional haemostatic measures.

Statistical analyses on blood loss during the operation, blood loss after cross-clamp removal, duration of operation, drain volume, requirement for blood transfusion and surgeons rating of efficacy were performed. Blood loss during operation was measured by using cell saver device (Dideco Electa, Sorin^® ^Group, Italy) and blood loss after cross-clump removal was measured by weighing the swabs used in the relevant wound from the time TachoSil^® ^or compresses were applied until achieving haemostasis. Duration of the operation was measured from the time of incision to the completion of skin suture. The drainage was directly related to the suture and its volume was evaluated at the time of removal after 36 hours. Surgeons rating of hemostasis efficacy was grade from 3 to 0: 3-"very good"; 2 - "good"; 1 - "satisfactory" and 0 - "unsatisfactory". All the variables (time to haemostasis, blood loss during the operation, duration of operation, drain volume, requirement for blood transfusion and surgeons rating of efficacy) of the two treatment groups were analyzed using SAS ^®^software (Version 9.2, SAS institute Inc., Cary NC, USA). Wilcoxon rank sum test was used to evaluate the association between each variable in the two treatment Group. Graft sizes were recorded. In 18 cases a straight Dacron graft tube was used, in 2 cases a bifurcated graft. One bifurcated graft was used in the Group I and the other one in Group II. All patients underwent same antibiotic protocol.

## Results

Ten patients were randomized in each treatment group of the study. In Group I, one patch was used in 5 patients, two patches in 4 patients and three patches in 1 patient. Postoperative clotting parameters were not altered by application of the patches. There were no instances of intravascular thrombosis or embolism subsequent to the application of TachoSil. The mean time to haemostasis was 264 ± 127.1 s (range: 180-600 s) in Group I and 408 ± 159.5 s (range: 120-720 s) in Group II. The Wilcoxon test based on the 20 patients with assessment of time to haemostasis showed statistical significance (p = 0.026). Complete haemostasis was achieved within 3 min in 4/10 patients (40%) in Group I and 1/10 patients (10%) in Group II. In Group I other 5/10 patients (50%) achieved haemostasis at the second look after 5 min making a total of 90% haemostasis after 5 min. In Group II 2/10 more patients (20%) achieved complete haemostasis at the second look adding to a total of 30% haemostasis after 5 min. Haemostasis was achieved within 5 min in 90% of the patients in Group I compared to only 70% within 7 min in Group II. The mean blood loss during the operation was 503.5 ± 20.7 cc (range: 474-545 cc) in Group I and 615.7 ± 60.3 cc (range: 530-720 cc) in Group II with significant statistical difference (p < 0.001). Mean blood loss after cross-clamp removal was 26.5 ± 4 g (range: 22-34 g) in Group I and 45.4 ± 4.6 g (range: 38-52 g) in Group II (p < 0.001). Mean time of operation was 111.7 ± 15 min (range: 99-150 min) in Group I and 119.9 ± 20.7 min (range: 95-170 min) in Group II and showed no statistical difference (p = 0.199). Mean drainage volume at 36 hours was 116.7 ± 41.4 cc (range: 79-230 cc) in Group I and 134.5 ± 42.8 cc (range: 101-250 cc) in Group II (p = 0.034). Perioperative blood transfusion was 1.3 units (range: 0-2 units) in Group I and 1.4 units (range: 0-3 units) in Group II (p = 0.968) (Table 2).

**Table 2:** Variables measured.

**Variable**	**TachoSil^® ^(n = 10)**	**Control (n = 10)**	**P**
Time to haemostasis (s)	264.0 ± SD 127.1	408 ± SD 159.5	0.026
Blood loss during the operation (cc)	503 ± SD 20.7	615 ± SD 60.3	<0.001
Blood loss after cross-clump removal (g)	26.5 ± SD 4	45.4 ± SD 4.6	<0.001
Duration of operation (min)	117 ± SD 15	119.9 ± SD 20.7	0.199
Drain volume (cc)	116.7 ± SD 41.4	134.5 ± SD 42.8	0.034
Requirement for blood transfusion	1.3 ± SD 0.8	1.4 ± SD 0.8	0.968
Surgeons rating of efficacy	2.6 ± SD 0.5	2.2 ± SD 0.8	0.257

SD: standard deviation; n: numbers of patients; sec: seconds; cc: cubic centimetres; g:grams; min: minutes.

No fever and allergic reaction to TachoSil^® ^components was registered in the two arms. In the sixth post-operative day, we registered, in Group II, a case of myocardial infarction treated with medical therapy. One case of renal failure requiring dialysis was seen in Group II. The non-serious adverse events were equally distributed between the two treatment groups. No adverse events were considered related to the test treatment.

## Discussion

TachoSil^® ^patches may be effective in delivering drugs locally. In this study TachoSil^® ^was used to provide fibrinogen and thrombin locally at the site of bleeding. Upon contact with fluid the clotting factors of TachoSil^® ^dissolve and form a fibrin network, which glues the collagen sponge to the wound surface. Combining the clotting factors in a collagen patch provides a high concentration of clotting factors at the site where it is specifically needed. The haemostatic effectiveness of using TachoSil^® ^has previously been proved in clinical studies [5-7]. Liquid fibrin glue preparations are likewise found to be effective in achieving haemostasis [8-10]. Other attempts used to control suture hole bleeding are ethylcyanoacyrlate glue, different forms of collagen, oxidized cellulose, topical thrombin and gelatine sponge or fibrin [11]. However, combining fibrin glue components in a collagen patch facilitates ease of application. Recently TachoSil^® ^patches was found significantly superior compared to conventional haemostatic fleece material for control of bleeding in cardiovascular surgery [12]. Despite the small number of patients enrolled, the present study showed that TachoSil^® ^significantly reduces the time to haemostasis. In addition both intra and postoperative bleeding were substantially reduced in the Group I. TachoSil^® ^was well tolerated and none of the adverse events observed in Group I were considered related to test treatment.

## Conclusion

In our experience, TachoSil^® ^was found to be safe and effective for the control of suture-hole bleeding in patients undergoing vascular reconstruction with Dacron grafts. Further studies with larger sample size are required to confirm the efficacy of TachoSil^® ^patches in controlling Dacron suture-hole bleeding after AAA open repair. Moreover, TachoSil^® ^could be proven as an useful tool in specific conditions such as coagulopathy and redo aortic surgery.

## List of Abbreviations used

cm: centimeters; UI: International Units; mg: milligrams; min: minutes; s: seconds; cc: cubic centimeters; g: grams; Kg: kilograms.

## Competing interests

The authors declare that they have no competing interests.

## Authors' contributions

GB has made substantial contributions to conception and design; he has given final approval of the version to be published. FP has made substantial contributions to acquisition, analysis and interpretation of data; he has been involved in drafting the manuscript. DM has made substantial contributions to acquisition, analysis and interpretation of data; he has been involved in drafting the manuscript. All authors read and approved the final manuscript.

## Authors' informations

GB: Professor of Vascular Surgery at University of Palermo.

Direttore Cattedra di Chirurgia Vascolare - University of Palermo.

Direttore Scuola di Specializzazione in Chirurgia Vascolare - University of Palermo.

Direttore U.O.C di Chirurgia Vascolare - A.O.U.P. 'Paolo Giaccone.'

FP: Fellow Vascular and Endovascular Surgery Unit - University of Palermo.

DM: Fellow Vascular and Endovascular Surgery Unit - University of Palermo.
